# Enhancement of Heat Stability and Kinetic Parameters of the Maize Endosperm ADP-Glucose Pyrophosphorylase by Mutagenesis of Amino Acids in the Small Subunit With High B Factors

**DOI:** 10.3389/fpls.2018.01849

**Published:** 2018-12-12

**Authors:** Susan K. Boehlein, Janine R. Shaw, L. Curtis Hannah

**Affiliations:** ^1^Genetics Institute, University of Florida, Gainesville, FL, United States; ^2^Department of Horticultural Sciences, University of Florida, Gainesville, FL, United States; ^3^Plant Molecular and Cellular Biology, University of Florida, Gainesville, FL, United States

**Keywords:** AGPase (ADP-glucose pyrophosphorylase), yield loss at harvest, heat lability, climate change, B-factors, protein evolution

## Abstract

ADP-glucose pyrophosphorylase (AGPase) is an important enzyme in starch synthesis and previous studies showed that the heat lability of this enzyme is a determinant to starch synthesis in the maize endosperm and, in turn, seed yield. Here, amino acids in the AGPase endosperm small subunit with high B-factors were mutagenized and individual changes enhancing heat stability and/or kinetic parameters in an *Escherichia coli* expression system were chosen. Individual mutations were combined and analyzed. One triple mutant, here termed *Bt2-BF*, was chosen for further study. Combinations of this heat stable, 3-PGA-independent small subunit variant with large subunits also heat stable yielded complex patterns of heat stability and kinetic and allosteric properties. Interestingly, two of the three changes reside in a protein motif found only in AGPases that exhibit high sensitivity to 3-PGA. While not the 3-PGA binding site, amino acid substitutions in this region significantly alter 3-PGA activation kinetics.

## Introduction

The heterotetrameric enzyme, ADP-glucose pyrophosphorylase (AGPase; EC 2.7.7.27), catalyzes the formation of PPi and ADP-glucose (ADP-Glc) from ATP and glucose-1-phosphate (G-1-P). The glucose of ADP-Glc is then used for starch synthesis. AGPase is an important control point in starch synthesis and checkpoints in the control of AGPase include allostery, transcription and posttranslational modification (reviewed in Ballicora et al., 2003; Hannah, 2007; Hannah and Green, 2008; Hannah and James, 2008; Preiss, 2009). Heat lability is a particularly important parameter for AGPases expressed in the endosperm (Preiss et al., 1971) since past studies point to grain loss from hot weather being linked to the heat lability of this enzyme (Greene and Hannah, 1998; Hannah et al., 2012, 2017). While heat lability of AGPase might have been evolutionarily desirable for the perpetuation of the species through the ages (Greene and Hannah, 1998), it is not ideal for maize kernel yield.

The relevance of AGPase to starch synthesis and, in turn, plant yield and plant agriculture is evident from eleven reports from six laboratories showing that placement of enhanced AGPases in important crop species increased starch content and, in turn, yield. Elevated yields from plants containing an altered AGPase have been reported in maize (Giroux et al., 1996; Wang et al., 2007; Li et al., 2011; Hannah et al., 2012, 2017), rice (Smidansky et al., 2003; Sakulsingharoj et al., 2004), wheat (Smidansky et al., 2002), potato (Stark et al., 1992; Lee et al., 2009), and Arabidopsis (Obana et al., 2006).

In this study, nine amino acids in the maize endosperm small subunit (BT2) with high predicted B-factor scores were mutagenized and variants screened for elevated activity. Amino acid substitutions at five of these sites gave rise to AGPase activity at 55°C; conditions that totally abolish activity of wild type maize endosperm AGPase. Changes at three sites conditioned 3PGA independent activity, while alterations at five sites lowered the 3PGA *K*_a_. Genes were synthesized containing multiple changes and one variant termed BT2-BF harboring three of these changes was chosen based on activity at 55°C and 3PGA independent activity.

Interestingly, two of the sites in BT2-BF correspond to two of the sites independently selected in the SH2-ISM variant described previously (Boehlein et al., 2015). SH2-ISM contains the changes Q96G; D161G; A443R. Hence, while the two subunits have diverged in function, motifs for enhanced heat stability have been maintained in the respective subunits through evolution.

Thirteen kinetic parameters were determined in AGPases containing BT2-BF expressed with *Sh2* variants that also alter heat stability and 3PGA independent activity. Resulting data were complex; synergistic interactions were observed in some cases, while in other cases, the change only needed to be present in one subunit to alter the final enzyme. Surprisingly, some alterations were antagonistic, resulting in an enzyme more closely resembling the wild type enzyme. Of particular significance, these studies, when coupled to previous investigations, point to a protein motif present only to AGPases exhibiting significant sensitivity to the activator 3-PGA. While this motif is not the binding site for 3-PGA, amino acid substitutions in this region significantly affect 3-PGA activation.

## Materials and Methods

### Plasmid Preparation

Saturation mutagenesis was performed on the *Escherichia coli* expression plasmid for the maize endosperm AGPase small subunit, pMONc*Bt2*, at the nine amino acid sites with high B- factors identified previously (Boehlein et al., 2015) (Table 1). Mutants were prepared using the Gibson Assembly Cloning Kit (NEB) to combine PCR products with the vector. PCR products for single site mutants were prepared using pMONc*Bt2* as template. Products for multiple site mutants were prepared from templates containing one or more of the mutant sites. The resulting mutant plasmids were co-expressed with the large subunit wild type plasmid, pMONc*Sh2*, or *Sh2* variants as specified below in the *glgC*^-^ mutant *E. coli* AC70R1-504 as previously described (Boehlein et al., 2008).

**Table 1:** Maize endosperm AGPase residues predicted to have unusually high relative B-factors by analogy with the potato small subunit homotetramer x-ray crystal structure (Jin et al., 2005).

Potato tuber ss residue	Residue mean B-factor*^a^*	Maize ls (Sh2) equivalent	Maize ss (Bt2) equivalent
Arg 33	62.36	Gln 96	Arg 57
Glu 99	74.38	Asp 161	Glu 123
Asp 218	71.25	Ile 285	Asp 242
Lys 222	68.41	Gln 289	Asp 246
Arg 307	65.10	Pro 372	Arg331
Asp 375	74.12	Glu 440	Glu399
Arg 378	67.55	Ala 443	Lys402
Lys 379	67.03	Ser 444	Lys403
Leu 380	66.24	Lys 445	Leu404

*^a^The average mean B-factor for all residues is 34.1.*

### Library Screening

Libraries were screened using the iodine staining method described in Boehlein et al. (2015). Briefly, transformed colonies grown overnight at 37 and 41°C on plates containing glucose and antibiotics were stained under iodine vapors for 1 min and scanned. The darkest staining colonies were selected and grown in liquid culture overnight at 37°C. The overnight cultures were spotted (1.5 μl) on two identical glucose plates and grown at 37 and 41°C overnight. Spots exhibiting darker stain than wild type were selected for sequencing.

### Enzyme Purification

The enzymes were expressed and purified using protocols developed in Boehlein et al. (2008).

### Catalytic Activity at 37 and 55°C

An end point assay was used to determine the amount of PPi produced from a reaction containing 50 mM HEPES pH 7.4, 15 mM MgCl_2_, 5 mM ATP, 2 mM G-1-P, 2.5 mM 3-PGA and 0.2 μg of purified enzyme. Reactions proceeded for 2, 4, 6, 8, 10, and 15 min and were terminated by boiling. Reaction mixes (1300 μl) were pre-warmed to 37°C or 55°C and the assay was started with the addition of the enzyme. Assay time points (200 μl) were removed at the appropriate time and boiled for 2 min. At 37°C all reactions were linear for 15 min; however, at 55°C rates were linear for a shorter duration. A rate was only calculated if the reactions were linear for at least 10 min.

The amount of PPi produced in the reaction was coupled to a decrease in NADH concentration. Coupling reagent contained 25 mM imidazole pH 7.4, 4 mM MgCl2, 1 mM EDTA, 0.2 mM NADH, 0.725 U aldolase, 0.4 U triose phosphate isomerase, 0.6 U glycerophosphate dehydrogenase, 1 mM fructose 6-phosphate and 0.8 μg purified PPi-PFK per reaction. Blank samples contained complete reaction mixtures minus the enzyme. Change in absorbance between the blank and the reaction was used to calculate the amount of PPi produced. All reactions were linear with time and enzyme concentration.

### Determination of Kinetic Constants, *K*_m_, *k*_cat_, and *K*_a_

The Michaelis constants for the substrates of the various proteins were determined by incubating the purified AGPase with a varying level of substrate (ATP or G-1-P) at a constant saturating level of co-substrate. All *V* versus *S* plots were fitted using non-linear regression analysis and the following equation, *v* = *V*_max_*S*/(*Km* + *S*), where *v* is the measured velocity, *V*_max_ is the maximum velocity, *S* is the substrate concentration. When held constant, reaction mixtures contained 50 mM HEPES pH 7.4, 15 mM MgCl2, 2.5 mM 3-PGA and 2.0 mM ATP or 2.0 mM G-1-P in a total volume of 200 μl. When varied, ATP and G-1-P concentrations were 0.05–3.0 mM and 0.025–1.5 mM, respectively. All saturation plots were hyperbolic. No cooperative effects were seen.

Likewise, the activation constant (*K*_a_) was determined by varying the activator (0–1 mM) at fixed levels of both substrates (2 mM). All reactions were developed as above. The activation constant was fitted to the following equation, *V* = *V*_min_ + *V*_max_^∗^*X*/(*K*_a_ + *X*), since several enzymes had appreciable activity in the absence of activator. Here, *V*_min_ is the velocity in the absence of activator and *V*_max_ is the change in activity from *V*_min_ to the total velocity, *X* is the activator concentration, *K*_a_ is the activation constant. All linear regressions were carried out using the software program Prism (GraphPad, San Diego, CA, United States).

Kinetic data for the determination of the *K*_m_ value in the absence of 3-PGA were fitted to equations using the non-linear equations and GraphPad Prism 4.0c. Initial velocity data were fitted to Eq. (1), which describes a sequential mechanism where *v* is the measured reaction velocity, *V* is the maximal velocity, *A* and *B* are the concentrations of substrates (ATP and G-1-P), *K*_a_, and *K*_b_ are the corresponding Michaelis–Menten constants, and *K*_ia_ is the dissociation constant for substrate *A*. The concentration of ATP was varied from 0.01–10 mM to 0.1–25 mM for G-1-P.

(1) $$v=VAB/(K_{\text{ia}}K_{{\text{m}}_{\text{b}}}+K_{{\text{m}}_{\text{b}}}A+K_{{\text{m}}_{\text{a}}}B+AB$$

### Pi Inhibition

Pi inhibition was carried out in both the presence and absence of 3-PGA with the Pi and substrate concentrations listed below.

**Table:** 

Varying substrate	Activator (3-PGA) (5 mM)	Substrate concentration when varied (mM)	Pi concentration when varied (mM)	Substrate concentration when held constant (mM)
ATP	–	0.1–3	10–30	1
G-1-P	–	5–30	5–50	5
ATP	+	0.025–1.5	0.5–25	0.4
G-1-P	+	0.025–1.5	1–25	0.2

### Data Analysis

Inhibition data were fitted to equations 2–3, which correspond to partial mixed type inhibition (Eq. 2), linear mixed type inhibition (Eq. 3) using GraphPad Prism software. v is the measured velocity, *V*_m_ is the maximum velocity, *S* is the substrate concentration, *K*_i_ is the inhibition constant, *K*_s_ is the dissociation constant for the ES complex, I is the inhibitor concentration, α is the factor by which *K*_i_ changes when the inhibitor is present and β is the factor by which *k*_p_ changes when the inhibitor is present. In a mixed type inhibition model, which includes competitive, non-competitive and uncompetitive models as special cases, an α value of 1 indicates that the effector does not alter the binding of the substrate and the mechanism reduces to a non-competitive model. A large α value indicates competitive inhibition whereby the inhibitor prevents binding of the substrate. If the inhibitor enhances the binding of the substrate, α is very small, and the model reduces to an uncompetitive model. An additional term (β) is added to the equations if the inhibition is not complete (high concentrations of the inhibitor do not drive the reaction rate to zero) and reflects the degree of inhibition. If both substrate binding (mixed type) and product formation (partial) are altered by the inhibitor, it is classified as partial mixed type inhibition. Special cases include pure non-competitive inhibition, where α = 1 and β = 0 and partial non-competitive inhibition whereby α = 1 and 0 < β < 1.

(2) $$v=V_{\text{m}}(\text{S}/K_{\text{s}}+(\beta \text{SI)/(}\alpha {\text{K}}_{\text{i}}{\text{K}}_{\text{s}}))/(1+\text{S/}K_{\text{s}}+\text{I}/K_{\text{i}}+\text{SI/}\alpha K_{\text{i}}K_{\text{s}})\text{P-MT}$$

(3) $$v=V_{\text{m}}(\text{S}/K_{\text{s}})/(1+\text{S}/K_{\text{s}}+\text{I/}K_{\text{i}}+\text{SI/}\alpha K_{\text{i}}K_{\text{s}})\text{L-MT}$$

While conclusions are drawn from these equations, a graphical representation of these plots can be found at https://chem.libretexts.org/Textbook_Maps/Biological_Chemistr y/Catalysts/Enzymatic_Kinetics/Enzyme_Inhibition.

## Results

Nine random replacement libraries were constructed involving amino acids substitutions in positions R57, E123, D242, K246, R331, E399, K402, K403, and L404. The thermal stabilities of resulting AGPase variants were then assessed from *E. coli* and resultant glycogen accumulation following *E. coli* growth at elevated growth temperatures (37 and 41°C). Transformants were grown in the presence of glucose and the accumulated glycogen was revealed by exposing bacterial colonies to iodine vapor. Both a wild type and a thermostable variant (MP-TI) AGPase (Boehlein et al., 2005; Georgelis and Hannah, 2008) were included as negative and positive controls, respectively. Digital photographs of plates were analyzed quantitatively by the ImageJ program to measure relative color density for each colony. Darker staining colonies were selected and their DNA sequences determined (Table 2). Three amino acid replacements conditioning darker staining colonies were found at each of the sites 123, 403, 404, while two were found at site 331, and single changes were found at positions 402, 242. None of the replacements at positions 57, 246, and 399 increased glycogen production. All selections sequenced for positions 57 and 399 contained altered codons that encoded the wild type amino acid.

**Table 2:** AGPase activity at 37 and 55°C in *Bt2* variants with substituted amino acids.

Mutant	Activity at 55°C	Activity at 37°C	% 55°/37°
wt	-	5415 ± 268	–
E123V	261 ± 32	5927 ± 190.4	4.4
E123G	1325 ± 172	7923 ± 410	16.7
E123Q	-	3080 ± 247	–
D242H	0	5583 ± 144	–
R331K	6822 ± 353	11543 ± 918	59.1
R331P	0	3132 ± 315	–
K402R	2702 ± 103.1	10738 ± 386	22.1
K403L	0	4555 ± 528	–
K403E	1970 ± 205	6279 ± 158	31
K403R	-	4446 ± 72	–
L404K	3774 ± 204	11985 ± 1862	23
L404E	2284 ± 236	9980 ± 356	21
L404H	9152 ± 903	12889 ± 3620	43
E123G, R331K, K402R, K404H	2349 ± 268	6150 ± 560	38.2
E123G, R331K, K402R (BT2-BF)	4555 ± 242	12163 ± 327	37.4
E123G, R331K, L404H	2942 ± 79	7732 ± 289	38
E123G, R331K	858 ± 222	4340 ± 158	19.8
SH2-E	6849 ± 845	6314 ± 648	111
SH2-ISM	4341 ± 451	7441 ± 122	82
^∗^SH2-E/BT2-BF	5192 ± 223	7552 ± 472	69
^∗^SH2-ISM/BT2-BF	6126 ± 560	12018 ± 392	51

*Enzyme activity was determined at saturating substrate concentrations in the presence of 2.5 mM 3-PGA for up to 15 min. ^∗^Double subunit mutants were linear for at least 45 min at 37 and 55°C.*

Each variant was expressed in *E. coli* with the wild type SH2 protein, resulting AGPase was purified and kinetic and allosteric properties were determined. Activity was determined at 37 and 55°C to identify mutations eliciting a more heat stable AGPase. The wild type AGPase is not active at 55°C. Two of the three E123 changes showed considerable activity at 55°C (Table 2). Compared to activity at 37°C, R331K retained ∼59% activity at 55°C, while a change to proline at this position had no detectable activity at 55°C even though glycogen staining of this variant was darker after growth at 41°C. The D242 mutant did not confer heat stable activity. However, each of several changes to amino acids 402, 403, and 404 conditioned considerable activity at 55°C. Thus, most of the darker staining colonies conferred a heat stable activity.

The Michaelis constants for ATP and G-1-P in the presence of 3PGA were determined. The *K*_m_ values for both ATP and G-1-P of all variants were similar to the wild type enzyme (Supplementary Table S1), 0.075 mM and 0.012 mM for ATP and G-1-P, respectively.

The 3PGA activation constant (*K*_a_) was also determined (Table 3). Two of the E123 variants had activity in the absence of 3PGA as did R331K and D242H.

**Table 3:** 3-PGA Ka values and activity in the presence and absence of 3-PGA in *Bt2* variants with substituted amino acids.

Mutant	*K*_a_^∗^	*V*_min_^∗∗^	*V*_max_^∗∗^
wt	0.26 ± 0.061	-	6352 ± 540
E123V	0.10 ± 0.036	1436 ± 465	6384 ± 585
E123G	0.090 ± 0.023	1646 ± 330	5883 ± 407
E123Q	0.12 ± 0.022	-	3674 ± 192
D242H	0.077 ± 0.0077	649 ± 172	6395 ± 194
R331K	0.07 ± 0.008	1420 ± 294	10664 ± 332
R331P	0.094 ± 0.013	-	3790 ± 127
K402R	0.077 ± 0.014	-	8568 ± 251
K403L	0.41 ± 0.049	-	7614 ± 334
K403E	0.14 ± 0.015	-	3295 ± 98
K403R	0.22 ± 0.057	-	5269 ± 475
L404K	0.14 ± 0.047	-	7082 ± 592
L404E	0.15 ± 0.014	-	8457 ± 226
L404H	0.05 ± 0.008	-	10899 ± 317
E123G, R331K, K402R, K404H	0.11 ± 0.017 +	538 ± 2231	6698 ± 260
E123G, R331K, K402R (BT2-BF)	0.060 ± 0.0097	2141 ± 356	8714 ± 410
E123G, R331K, L404H	0.059 ± 0.11		4978 ± 241
E123G,R331K	0.183 ± 0.024	1167 ± 125	5864 ± 187
SH2-E	0.070 ± 0.01	1183 ± 177	4679 ± 750
SH2-ISM	0.056 ± 0.008	1150 ± 173	8850 ± 850
SH2-E/BT2-BF	0.36 ± 0.09	969 ± 52	2361 ± 183
SH2-ISM/BT2-BF	0.23 ± 0.11	2827 ± 165	2603 ± 338


*^∗^The activation constant, K_a_, was determined for each of the enzymes in the presence of 2 mM ATP and 2 mM G-1-P. ^∗∗^V_min_ refers to the activity in the absence of 3-PGA. V_max_ here is the activity gained by the addition of 3-PGA.*

Individual amino acid changes conditioning activity at 55°C and/or 3PGA independent activity were combined and the properties of the stacked variants were then examined. Previously we were successful using a similar combinatorial method with sites selected from evolutionary considerations (Boehlein et al., 2014). Variant sites were chosen by the following rationale: of the E123 substitutions, E123G had the greatest activity at 55°C. R331K was selected because of elevated activity at 55°C and 3PGA independent activity. The only mutation at position 242 had comparable activity to wild type, and hence was not included in the final construct. The K402R mutation was chosen because it had activity at 55°C. L404H was chosen since it had the highest activity at 55°C.

The quadruple variant was constructed, expressed, and resulting properties are given in Tables 2, 3. While this variant exhibited activity at 55°C, activity at 37°C was much less than some of the single mutant counterparts. Two sites, 402 and 404 are in close proximity, suggesting the possibility of unexpected interactions. Accordingly, the variants E123G and R331K were combined and then K402R and L404H were added individually. E123G with R331K had approximately the same activity as the quadruple mutant (Tables 2, 3); whereas, the triple combination containing K402R had almost double the activity of the quadruple variant and the other triple combination containing L404H. Thus, the resulting triple variant containing the E123G, R331K, and K402R mutations, now termed BT2-BF (for B-factors) was further characterized and compared to our previously constructed heat stable clones.

Activity in the absence of the activator 3PGA was examined. While 3PGA is a noted activator for plant AGPase, the mechanism of activation differs for the various AGPases. In some cases, it does not activate, but rather relieves Pi inhibition. In other cases, for example the maize endosperm AGPase, it lowers the *K*_m_ for ATP or G-1-P. Thus, deciphering the kinetics in the absence of 3PGA allows us to draw various conclusions (Boehlein et al., 2013). ATP and G-1-P *K*_m_s in the presence of 3PGA are 0.075 and 0.012 mM (Supplementary Table S1) for the wild type maize endosperm AGPase while in the absence of 3PGA the values are ∼4.0 and 2.3 mM, respectively. Thus, the binding of 3PGA reduces the *K*_m_ values of both substrates over 50-fold (Table 4). While there are various reports of “3PGA activating” the enzyme at single fixed substrate concentrations, this “activation fold” will vary with substrate concentration if the mechanism of 3PGA activation involves a change in the *K*_m_ for the substrate. Thus, the term “activation fold” in the literature is merely an activity reported at single fixed substrate concentrations. These concentrations are usually saturating based on the *K*_m_ values reported in the presence of 3PGA but not necessarily saturating in the absence of 3PGA. For example, when comparing activation fold for the potato tuber enzyme and the maize endosperm enzyme at 1 mM of each substrate, the maize endosperm activity would be greatly enhanced since the *K*_m_s in the absence of 3PGA are 4 and 3 mM, respectively, for ATP and G-1-P. Thus, addition of 3PGA would reduce the *K*_m_s to saturating levels and velocity would increase. In contrast, 3PGA does not decrease the *K*_m_ values for ATP and G-1-P (Boehlein et al., 2013) for the potato tuber enzyme. In this scenario, the potato tuber “activation fold” would also be affected, but for completely different reasons. The *K*_m_ values for ATP and G-1-P are comparable regardless of inhibitor concentration, but the activator increases *k*_cat_ by ∼10-fold. Thus, both enzymes would be activated by 3PGA, but by different mechanisms. Hence, reports in the literature of activation folds measured at single substrate concentrations (for example, pea, Hylton and Smith, 1992; Vicia faba seeds, Weber et al., 1995; and maize endosperm, Plaxton and Preiss, 1987) without an understanding of the mechanism of activation must be interpreted carefully.

**Table 4:** Kinetic values determined in the absence of 3-PGA of various AGPases.

Mutant	ATP *K*_m_	G-1-P *K*_m_	*K*_i_s (ATP)
wt	a4.0 ± 0.35	2.8 ± 2.1	nd
MP	a0.9 ± 0.4,	11.9 ± 3.0	nd
	b0.43 ± 0.04
SH2-E	2.5 ± 0.43	0.03 ± 0.005	5.4 ± 5.6
SH2-ISM	0.1 ± 0.048	8.7 ± 1.2	0.7 ± 0.15
BT2-BF	0.30 ± 0.25	4.1 ± 1.5	5.1 ± 2.3
SH2-E/BT2-BF	2.41 ± 0.35	0.038 ± 0.009	3.17 ± 1.63
SH2-ISM/BT2-BF	0.51 ± 0.10	0.023 ± 0.008	1.59 ± 0.74
MP + SH2-E	0.32 ± 0.021	0.09 ± 0.008	0.59 ± 0.74

*a ATP k_m_ could not be determined because G-1-P was not saturating, the value reported is the apparent K_m_ in the presence of 15 mM G-1-P. b in the presence of 25 mM G-1-P. K_i_s-dissociation constant for the E-ATP complex.*

Previously, we constructed several heat tolerant AGPases. One involved amino acid substitutions in the small subunit (Boehlein et al., 2005), while three involved changes in the large subunit (Greene and Hannah, 1998; Boehlein et al., 2014, 2015). The data are summarized in Table 4 for the small subunit variant and two of the large subunits variants. The small subunit mutant, MP, contains the first 198 amino acids of the small subunit of the maize endosperm enzyme and the last 277 amino acids from the potato tuber enzyme resulting in thirty-three amino acid sites that differ from the maize endosperm enzyme (Cross et al., 2004). When co-expressed with the wild type large subunit (SH2) the resulting AGPase exhibits ∼10-fold reduction in the ATP *K*_m_ in the absence of 3PGA. In fact, it approaches the ATP *K*_m_ for the wild type in its activated state. Saturating concentrations of G-1-P could not be obtained for the MP variant, so the true *K*_m_ for ATP could not be calculated, but at 25 mM G-1-P, the apparent ATP *K*_m_ was 0.43 mM.

The SH2-E variant having the changes N131R; T142F; A160T; Q261S; A396S; V416I; C424V; S444A was synthesized based on evolutionary considerations while SH2-ISM arose from structural considerations. Both variants produced a heat stable AGPase when expressed with the BT2 subunit. In the un-activated state (-3-PGA), the wild type and SH2-E AGPases had similar, high ATP *K*_m_ values. In contrast, the G-1-P *K*_m_ for SH2-E was almost 100-fold lower than that of wild type, comparable to that of the activated form of the wild type enzyme (Table 4). Interestingly, the SH2-ISM construct yielded the opposite effect (Table 4). Here, the G-1-P *K*_m_ remained very high in the absence of 3PGA, while the ATP *K*_m_ was now comparable to that of the 3PGA activated state.

The triple small subunit variant described here, BT2-BF, had characteristics similar to SH2-ISM, in that the ATP *K*_m_ was reduced in the un-activated state, while the G-1-P *K*_m_ remained elevated.

Pi inhibition patterns in the presence and absence of 3PGA were also determined. Pi inhibition patterns were obtained by varying one substrate at several fixed concentrations of Pi, while holding the co-substrate at constant sub-saturating levels. These data were fitted to various mixed type inhibition equations as described in Section “Materials and Methods.”

Pi inhibition of AGPase containing the BT2-BF small subunit and the wild type SH2 (SH2-WT) large subunit (SH2-WT/BT2-BF) in the presence of 3PGA exhibits a linear mixed-type form of inhibition with respect to both ATP and G-1-P (Table 5). When G-1-P is the varied substrate, α is less than 1, indicating that the addition of Pi decreases the *K*_m_ value. This is not the case when ATP is the varied substrate. Interestingly, Pi inhibition of the wild type enzyme (SH2-WT/BT2-WT) only shows partial inhibition in the presence of 3PGA, thus increasing Pi never completely inhibits the enzyme. This is in contrast to the previously constructed heat stable variants containing the BT2-WT protein with SH2-E or SH2-ISM, which all show linear patterns, similar to SH2-WT/BT2-BF AGPase. Upon inspection, the Pi pattern of inhibition of SH2-WT/BT2-BF most closely resembles that of Sh2-ISM/BT2-WT. Hence, alteration of either subunit yields the same changed pattern of Pi inhibition.

**Table 5:** Pi inhibition in the presence of 3-PGA of various AGPases.

Large subunit	Small subunit	Varied substrate	Pattern	*K*_m_^∗^ (mM)	*K*_i_ (mM)	α	β
wt	wt	ATP	P-MT	0.11 ± 0.0057	0.37 ± 0.053	4.61 ± 0.07	0.83 ± 0.037
Sh2-ISM	wt	ATP	L-MT	0.11 ± 0.005	10.7 ± 1.7	2.5 ± 0.66	
SH2-E	wt	ATP	NC	0.21 ± 0.01	7.8 ± 0.21		
wt	BT2-BF	ATP	L-MT	0.20 ± 0.11	12.4 ± 1.9	1.48 ± 0.33	
SH2-E	BT2-BF	ATP	P-MT	0.23 ± 0.018	5.52 ± 1.23	0.69 ± 0.13	0.32 ± 0.028
SH2-ISM	BT2-BF	ATP	U	0.14 ± 0.009	52.0 ± 8.5		
wt	wt	G-1-P	P-MT	0.091 ± 0.0042	1.00 ± 0.18	0.90 ± 0.012	0.52 ± 0.023
Sh2-ISM	wt	G-1-P	NC	0.085 ± 0.003	20.7 ± 0.80		
SH2-E	wt	G-1-P	NC	0.063 ± 0.003	6.62 ± 0.39		
wt	BT2-BF	G-1-P	L-MT	0.17 ± 0.16	18.6 ± 6.0	0.38 ± 0. 14	
SH2-E	BT2-BF	G-1-P	U	0.16 ± 0.01	10.8 ± 0.93		
SH2-ISM	BT2-BF	G-1-P	U	0.31 ± 0.023	22.6 ± 2.6		

*^∗^K_m_ of varied substrate (G-1-P or ATP).*

In the absence of 3PGA, the pattern of Pi inhibition of SH2-WT/BT2-BF was similar to that of the SH2-ISM/BT2-WT AGPase (Table 6). Both showed linear kinetics with elevated *K*_i_s (≥30 mM) for Pi regardless of the varied substrate. The wild type and SH2-ISM/BT2-WT AGPases differ from the SH2-WT/BT2-BF enzyme in that the *K*_i_ for Pi was about 5–10-fold higher in the absence of 3PGA compared to that in the presence of 3PGA. Inhibition was still partial in the case of the wild type enzyme and complete (linear) for the SH2-ISM/BT2-WT variant. Pi inhibition of SH2-E/BT2-WT AGPase in the presence or absence of 3PGA was similar when ATP was the varied substrate. When G-1-P was the varied substrate, only partial inhibition occurred, but inhibition was seen at a very low concentration, (0.055 mM), over 100× less than in the presence of 3PGA.

**Table 6:** Pi inhibition in the absence of 3-PGA of various AGPases.

Large subunit	Small subunit	Varied substrate	Pattern	*K*_m_ (mM)	*K*_i_ (mM)	α	β
wt	wt	ATP	P-MT	20.4 ± 10.1	3.4 ± 1.1	0.063 ± 0.034	0.52 ± 0.17
Sh2-ISM	wt	ATP	NC	0.54 ± 0.057	113.0 ± 27		
SH2-E	wt	ATP	NC	0.97 ± 0.081	20.9 ± 1.6		
wt	BT2-BF	ATP	NC	0.79 ± 0.075	28.1 ± 5.0		
SH2-E	BT2-BF	ATP	U	4.98 ± 0.66	41.1 ± 9.4		
SH2-ISM	BT2-BF	ATP	P-MT	3.60 ± 1.63	0.88 ± 1.01	0.31 ± 0.18	0.97 ± 0.2
wt	wt	G-1-P	P-MT	22.2 ± 9.0	5.1 ± 2.1	0.095 ± 0.065	0.92 ± 0.31
Sh2-ISM	wt	G-1-P	NC	13.1 ± 0.96	52.7 ± 2.9		
SH2-E	wt	G-1-P	P-NC	0.20 ± 0.019	0.055 ± 0.013	0.85 ± 0.17	0.44 ± 0.03
wt	BT2-BF	G-1-P	U	46.08 ± 5.7	31.0 ± 13.8		
SH2-E	BT2-BF	G-1-P	P-MT	0.14 ± 0.02	0.22 ± 0.18	0.48 ± 0.11	0.68 ± 0.03
SH2-ISM	BT2-BF	G-1-P	P-MT	0.11 ± 0.017	1.71 ± 2.0	0.68 ± 0.2	1.14 ± 0.09

The BT2-BF small subunit was expressed in *E. coli* with each of the heat stable large subunits (SH2-E and SH2-ISM) and the catalytic properties of the resulting purified enzymes were determined. Activity in the absence of 3PGA (*V*_min_) and the additional activity gained by the addition of 3PGA (*V*_max_) were determined. *V*_min_ approximated *V*_max_ at 2 mM substrate concentrations for the SH2-ISM/BT2-BF enzyme while *V*_min_ only equaled 25% of *V*_max_ for the SH2-WT/BT2-BF enzyme (Table 3). To determine whether this reflected a change in *K*_m_ or *k*_cat_ values in the absence of the activator the *K*_m_ values for ATP and G-1-P in the absence of 3PGA were determined (Table 4). Compared to SH2-WT/BT2-BF, the SH2-ISM/BT2-BF AGPase had considerably lower *K*_m_ for G-1-P but a similar ATP *K*_m_. Thus, in the absence of the activator the double mutant SH2-ISM/BT2-BF is far superior to its single subunit counterpart (BT2-BF). A similar experiment using SH2-E/BT2-BF showed that the ATP *K*_m_ was elevated almost 10-fold from the single BT2-BF, while retaining a low G-1-P *K*_m_. Interestingly, in the absence of 3PGA the kinetics of the SH2-E/BT2-BF looked almost identical to the single subunit mutant SH2-E/BT2-WT.

Pi inhibition in the presence of 3PGA was then explored. When varying ATP in the presence of 3PGA, SH2-E/BT2-BF did not resemble SH2-E/BT2-WT or SH2-WT/BT2-BF. (Table 5). Both SH2-E/BT2-WT and SH2-WT/BT2-BF showed linear mixed type inhibition with relatively high *K*_i_ values for Pi (10–12 mM) while the double variant SH2-E/BT2-BF exhibited a partial mixed type inhibition with a slightly lower *K*_i_ for Pi (5.5 mM). The SH2-E/BT2-BF mutant is uncompetitive with respect to G-1-P, thus binding and product formation are altered by Pi in this variant. For SH2-ISM/BT2-BF, varying either ATP or G-1-P yield uncompetitive patterns with very high *K*_i_ values (22.6 and 55.2 mM for ATP and G-1-P, respectively).

Pi inhibition in the absence of 3PGA was also examined. SH2-E/BT2-BF AGPase yielded an uncompetitive pattern with respect to ATP and a partial mixed type inhibitor with respect to G-1-P, a pattern similar to SH2-E/BT2-WT where the *K*_i_ for Pi was extremely high with respect to ATP (28 and 41 mM for SH2-E/BT2WT and SH2-E/BT2-BF, respectively) and very low with respect to G-1-P (0.055 and 0.22mM for SH2-E/BT2WT and SH2-E/BT2-BF, respectively).

A different pattern was exhibited by the SH2-ISM/BT2-BF AGPase. Whereas the two single variants, SH2-ISM/BT2-WT and SH2WT/BT2-BF, significantly increased the *K*_i_ for Pi when ATP or G-1-P was varied, the combination of the two altered subunits gave rise to a *K*_i_ for Pi actually lower than that observed for the wild type (SH2WT/BT2-WT) enzyme.

AGPase activity at elevated temperatures was a criterion used in selecting in the individual substitutions of BT2-BF. Previously, we showed that the *Sh2* variants SH2-ISM and SH2-E when expressed with BT2-WT small subunit conditioned activity at 55°C that equaled 82% and 111%, respectively, of the activity detected at 37°C (Boehlein et al., 2014, 2015). Accordingly, we expressed the *Sh2* variants with BT2-BF (SH2-ISM/BT2-BF and SH2-E/BT2-BF, Table 2). While each of these combinations was active at 55°C, the extent of activity at 55°C relative to activity at 37°C was not increased relative to the amount conditioned by the single variants SH2-ISM and SH2-E.

## Discussion

Plant AGPases contain two small subunits and two large subunits in an α2β2 structure (reviewed in Keeling and Myers, 2010). While the subunits are encoded by separate genes, the proteins exhibit significant sequence identity and similarity and most likely arose via duplication from a common progenitor (Bae et al., 1990; Bhave et al., 1990). For example, the gene studied here, the maize endosperm small subunit encoded by the gene *brittle-2* (*Bt2*), exhibits 43.2% identity and 61% similarity with the endosperm cognate large subunit encoded by the *shrunken-2* (*Sh2*) gene. While of common origin, the functions of the small subunit and large subunit have diverged to the point that the subunits are not interchangeable. Both subunits are required for significant catalytic activity as well as for allosteric properties (Cross et al., 2004).

Because endosperm AGPases are heat labile and endosperm variants with enhanced heat stability give rise to enhanced yield, we used two predictive methods to identify variants in the large subunit that conditioned more heat stable maize endosperm AGPase. The first method was based on a phylogenetic approach, whereby type II and positively selected amino acids were changed and evaluated (Boehlein et al., 2014). Type II amino acids are invariant in gene family members expressed in one tissue, but variable in family members expressed in other tissues.

The second technique utilized iterative site-specific saturation mutagenesis of amino acids with high B-factor values predicted from the crystal structure of the potato tuber small subunit homotetramer (Boehlein et al., 2015). Highly mobile residues can be predicted from atomic displacement parameters (B-factors) in protein crystal structures. Since a crystal structure does not exist for the maize endosperm AGPase, the structure of a closely related AGPase, the potato small subunit homotetramer, PDB conde 1YP2 (Jin et al., 2005), was used to predict highly mobile amino acids. The rationale for the selection of the nine conformationally disordered residues found in surface loops of the potato small subunit was explained previously (Boehlein et al., 2015). Following mutagenesis and expression in *E. coli*, glycogen staining was used as the primary screen to determine if the changes enhanced or reduced enzyme function.

Data reported herein show that amino acid substitutions in the small subunit of the maize endosperm AGPase alter kinetic and allosteric properties as well as heat stability. These data are in accord with the hypothesis that both the small and large subunit are critical for both catalysis and allostery and provide further evidence questioning the idea that the subunits have distinctly different roles (Ballicora et al., 2003).

The variants described here have practical significance for plant yield. As described above, expression of the transgenes *Sh2-HS33/Rev6* (Hannah et al., 2012) or *Bt2-MP* (Hannah et al., 2017) in maize enhanced yield in hot environments. The two variants are more heat stable than their wild type counterparts. Hence, placement of the variants described here could increase maize yield.

The single amino acid changes described here affect protein stability, catalysis, and allostery. We have reported this pattern previously with other mutants in these genes and it is interesting that Rader and Brown (2011) reviewed evidence from other enzymes showing that the interdependence of these changes occurs through a rigid structure connecting domains for catalysis and allostery.

Interestingly, while the two subunits share a common origin, they have diverged over evolutionary time to be complementary rather than duplicate genes. Properties once contained in a single subunit for activity are now shared between the two different subunits. While divergent, here we identify amino acids at homologous positions in the two subunits that condition a common phenotype: heat stability and alteration in allosteric properties. Two of the three amino acid changes in BT2-BF correspond to two of the three amino acid changes in the SH2-ISM variant. (1) BT2 E123G and SH2 D161G and (2) BT2 K402R and SH2 A443R are homologous sites. Hence, at least for two motifs, one property is still shared between the two subunits.

**FIGURE 1:**
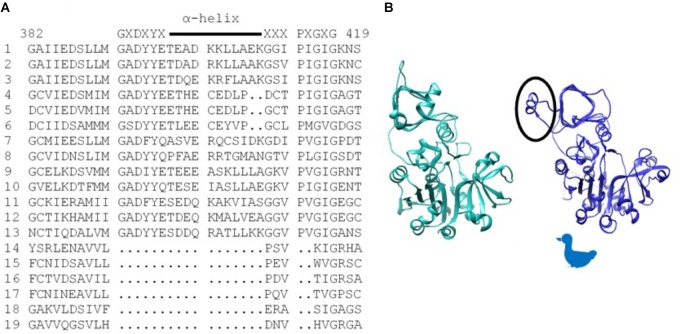
**(A)** Alignment of various AGPase sequences showing the sequence of the structure unique to chloroplast containing organisms. Consensus sequence is written above, and the α-helix is indicated with a line. Numbering is from the BT2 sequence. (1) Maize BT2, (2) Potato small subunit, (3) *M. sagu* agpll, (4) *M. pusilla* ADG1, (5) *M. commode* ADG1, (6) *C. reinhardtii* STA6, (7) *Anabaena*, (8). *N. sphagnicola*, (9) Maize SH2, (10) Potato large subunit, (11) *M. pusilla* ADG2A, (12) *M. commode* ADG2A, (13) *C. reinhardtii* STA1, (14) *A. tumefaciens*, (15) *E. coli*, (16) *A. ursilacus*, (17) *C. grimmiae*, (18) *Curtobacterium* sp., (19) *Streptomyces* sp. **(B)** *Agrobacterium* subunit (sea green) and potato small subunit (blue) highlighting (circled) the potato structure (GaG) protruding from the fourth loop of the p-helix. Because the subunit structure approximates the profile of a duck, the duck is used in subsequent figures to aid in understanding dimer and tetramer structures.

**FIGURE 2:**
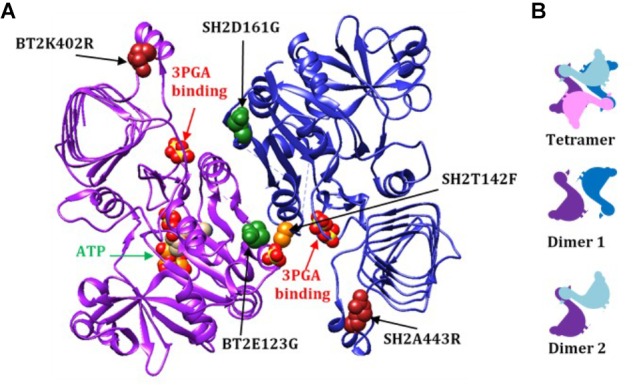
**(A)** A dimer from the AGPase crystal structure (dimer 1), viewed from the inside of the tetramer demonstrating how the two GαG motifs relate to the two 3PGA binding sites. The 3PGA binding sites are defined by the pair of sulfates from the crystal structure indicated by the red arrows. Here the subunits are designated BT2 (purple ribbon) and SH2 (dark blue ribbon). The ATP in the BT2 active site is indicated by the green arrow. Mutated sites are shown as colored spheres: BT2 E123G (dark green spheres in purple subunit), SH2 D161G (dark green spheres in blue subunit), SH2T142F (light orange spheres in blue subunit), BT2 K402R (dark red spheres in the purple subunit), and SH2 A443R (dark red spheres in the blue subunit). The spheres are from of the published potato crystal structure. **(B)** Rough two-dimensional diagrams of the tetramer and two dimers of small/large subunits. Small subunits are purple and pink and large subunits are dark and light blue.

Of particular significance, amino acid substitutions that alter 3PGA activation occur in a protein motif unique to AGPases that exhibit significant activation by this sugar acid. These reside in organisms that contain chloroplasts. Alignment of various AGPase sequences (Figure 1A) identified this motif which begins with the conserved amino acid sequence GXDXYX, followed by a nine to eleven amino acid sequence that forms an α-helix in the potato small subunit structure (Jin et al., 2005). The sequence in the α-helix varies in plant large and small subunits (Supplementary Figure S1). The motif returns to the β-helix with the conserved sequence XXXPXGXG. For simplicity, this motif is here termed the GαG structure. The last two amino acids of the motif (XG) align with IG in the *Agrobacterium* β-helix. Figure 1B shows the *Agrobacterium* crystal structure (Cupp-Vickery et al., 2008) and one subunit from the potato tuber small subunit homotetramer crystal structure (Jin et al., 2005) side by side with the GαG motif circled. In the crystalized bacterial enzyme, four amino acids occur in the position of this motif maintaining the shape of the β-helix. The GαG structure projects above or below the activator binding site (Boehlein et al., 2010) formed by the dimerization of one small subunit and one large subunit as depicted in Figure 2A.

Mutations in amino acids in the GαG α-helix alter affinity for the activator 3PGA, even though they are not part of the 3PGA binding site. Of the four mutations initially selected for the BT2-BF clone, K402R and L404H reside in this motif. Individual amino acid substitutions at each of these sites reduced the *K*_a_ for 3PGA. However, the two mutations together increased the *K*_a_. This is demonstrated in Tables 2, 3 from comparison of the double mutant BT2 E123G;R331K with the BT2-BF triple mutant. Addition of the K402R mutation decreases the *K*_a_ and increases activity with and without 3PGA and at both 37 and 55°C. Several other important sites have been documented that occur in the GαG α-helix (Cross et al., 2004; Boehlein et al., 2009, 2015; Georgelis et al., 2009; Boehlein et al., 2014). These are summarized in Table 7.

**Table 7:** Mutants located in or associated with the GαG domain.

BT2^a^	SH2^a^	Reference	Description
K402R	A443	This paper	Reduced *K*_a_ for 3-PGA, activity at 55°C
K402	A443R:	Boehlein et al., 2015	Stained darker at 37 and 42°C
K403	S444R	Boehlein et al., 2015	Stained darker at 37 and 42°, had higher activity at 37°
	S444A	Boehlein et al., 2014	Did not alter 3-PGA *K*_a_
L404H	K445	This paper	Reduced *K*_a_ for 3-PGA. Activity at 55°C
L404	K445R	Boehlein et al., 2015	Stained darker at 37 and 42°C
	E438Q	Georgelis et al., 2009	Did not alter 3-PGA *K*_a_
MP changes: E399D, K402R, E407A, G410S, I411V		Cross et al., 2004; Boehlein et al., 2009	Activity at 55°C, activity in the absence of 3-PGA
R331K	P372	This paper	Activity in the absence of 3-PGA
R331	P372A	Georgelis et al., 2009; Boehlein et al., 2015	Increased 3-PGA *K*_a_, decreased *K*_i_ for phosphate and reduced glycogen staining

*^a^When relevant, the cognate amino acid in the other subunit is listed.*

Analyses of the potato small subunit crystal structures show that the BT2-BF change R331K may affect the SH2 GαG structure through dimer 2 (Figure 2B) subunit:subunit interaction. The change of arginine to lysine reduced the *K*_a_ for 3PGA and conditioned 3-PGA independent activity. The SH2 homolog of BT2 R331, SH2 P372, was assessed in both the SH2 evolution (Georgelis et al., 2009) and ISM (Boehlein et al., 2015) mutagenesis experiments. Substitution of an alanine for this proline increased the 3PGA *K*_a_ (Georgelis et al., 2009).

**FIGURE 3:**
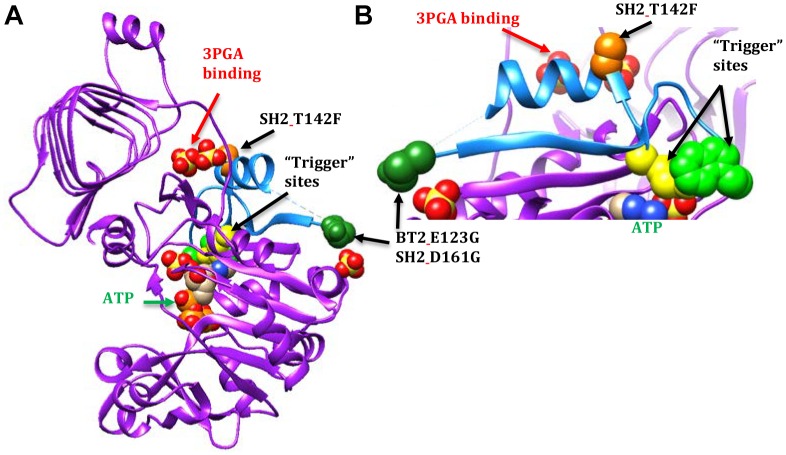
AGPase crystal structure diagrams with part of the Rossmann-like motif shown in light blue. A dashed line occurs between the dark green spheres and the blue a-helix because the amino acids in this area were too mobile to map in the potato crystal structure. In this particular subunit, nine amino acids are missing. The active site is indicated by the ATP (green text and arrow). The activator site is indicated by the two sulfates below the GaG motif (red text and arrow). The BT2 and SH2 amino acid sites of interest are represented by colored spheres: BT2.E123G/SH2 D161G (dark green spheres) and SH2.T142F (light orange spheres). The spheres are amino acids in the potato crystal structure. The *E. coli* “trigger” sites are represented by yellow spheres (Q74 or SH2_Q138) and light green spheres (W113 or SH2.W179). **(A)** A single subunit viewed from the inside of the tetramer. **(B)** Close-up of part of the Rossmann-like motif.

A second motif conferred 3PGA independent activity to AGPase (Figure 3). This motif is part of the Rossmann-like structure made of generally parallel β-strands separated by α-helices found in the N-terminal portion of the subunit. The section of interest here includes the β-strand and α-helix that occur between the two “trigger” sites described by Figueroa et al. (2011), *E. coli* Q74 and W113 (BT2 Q99 or SH2 Q138 and BT2 W140 or SH2 W179, respectively). Alteration of these sites did not affect activator binding; however, activity was not increased by the presence of the activator.

In contrast, 3-PGA independent catalysis occurs in the BT2-BF, SH2-ISM, and SH2-E variants. The BT2-BF mutant E123G and its homolog in SH2-ISM, D161G, are found in the center of the tetramer near one end of a β-strand (Figure 3). This β-strand traverses the subunit to a loop that moves with the binding of ATP (Jin et al., 2005). One of the amino acids in this loop is the *E. coli* “trigger” site W113. The SH2-E mutant T142F occurs in the α-helix adjacent to the 3PGA binding site. This α-helix is between the β-strand discussed above and another β-strand that traverses the subunit in parallel and includes the *E. coli* Q74 “trigger” site near the ATP binding site. All three of these mutations bestow activity in the absence of 3PGA, improved heat stability (activity at 55°C), and decreased *K*_a_ to the enzyme.

Combining BT2-BF with the heat stable large subunit mutants SH2-ISM (Q96G, D161G, A443R) and SH2-E (N131R, T142F, A160T, Q261S, A396S, V416I, C424V, S444A) resulted in additional insights. Activity at 37°C did not change appreciably but the ratio of 55°C/37°C did increase to over 50%. However, the 3PGA *K*_a_ reverted to wild type levels regardless of which altered large subunit was used. Replacement of the wild type SH2 subunit with SH2-E reduced the *V*_min_ and substitution with SH2-E or SH2-ISM reduced the *V*_max_. The ATP *K*_m_ was reduced for BT2-BF and SH2-ISM when expressed with their wild type counterparts and assayed in the absence of 3PGA. In the combination BT2-BF/SH2-ISM, the ATP *K*_m_ was reduced as in each individual variant, but, intriguingly, the G-1-P *K*_m_ was also reduced. In contrast, the G-1-P *K*_m_ was reduced in the absence of 3PGA for SH2-E expressed with the wild type BT2. SH2-E dominated the kinetics in the BT2-BF/SH2-E mutant combination. The G-1-P *K*_m_ was reduced while the ATP *K*_m_ was not. These perhaps unexpected differences may be due to the locations of the mutations in the 3PGA binding sites. A dimer formed by a large and small subunit creates two activator binding sites (Figure 2A). BT2-BF and SH2-ISM have mutations in the homologous sites BT2 E123G and SH2 D161G (dark green spheres in Fig 2A). Each of these sites is in a different activator binding site formed by the dimer. The tetramer is made up of two of these dimers resulting in four activator sites each containing an altered amino acid. Hence, for the BT2-BF/SH2-ISM enzyme, all four active sites can function independently of 3PGA with a lowered ATP *K*_m_. This combination also lowered the G-1-P *K*_m_ in the absence of 3PGA. The primary mutation in SH2-E that results in 3PGA-independent activity is T142F (light orange spheres in Figure 2A). This mutation lies in the same activator site as BT2 E123G and interacts with the same Rossmann-like motif in BT2. In SH2, the T142F mutation site is on the α-helix between the second and third β-strands of the SH2 Rossmann-like motif and thus may affect the activation of both subunits. This may negate the effect of the BT2-BF mutation.

This study combined with the results of earlier mutants has elucidated two key motifs of AGPase enzyme function. These motifs are found in both the large and small subunits since mutations at homologous sites have similar effects on the enzyme. Both motifs enhance heat stability of the enzyme and alter allosteric parameters in distinct ways.

The Rossmann-like motif in the N-terminal region, or at least part of the motif, appears to interface between the active and activator sites. The Rossmann fold motif occurs in nucleotide binding proteins and a similar motif is likely found in all AGPases. Comparison of the potato small subunit and *Agrobacterium* crystal structures shows that both contain a surprisingly similar pattern of Rossmann-like β-strands. Our mutants show that changes in this motif can condition 3-PGA independent activity although maximal activity requires the presence of 3-PGA.

The GαG motif in the C-terminal β-helix is found only in AGPases activated principally by 3PGA. Bacterial AGPases, activated by larger sugars such as fructose 6-phosphate, do not have this motif. This suggests that the motif may be involved in selection or efficiency of the activator. Fructose 6-phosphate can activate the maize wild type enzyme (Boehlein et al., 2008), but not as efficiently as 3PGA. Some mutants in the α-helix of this motif affect the 3PGA *K*_a_. It is not known whether these mutants effect activation by other compounds such as fructose 6-phosphate.

The interplay between heat stability and enzyme kinetics in AGPase is complex. The mutants discussed here were designed to increase the heat stability of the maize endosperm AGPase. The enzymes resulting from either BT2 or SH2 mutants with wild type counterparts had increased activation efficiency: reduced *K*_a_ and greater activity in the absence of activator. Enzymes resulting from combining the mutant subunits retained the heat stability (55°C activity) and 3PGA independent activity. However, the 3PGA *K*_a_s returned to wild type levels. Combining mutant subunits with superior activity in the *E. coli* expression system did not result in an enzyme with greater activity than the individual mutants.

## Conclusion

The studies reported here are significant at several levels. (a) The variants described here may have practical value since heat lability of the endosperm AGPase has been shown to reduce seed yield. (b) While the small and large subunit arose via gene duplication, sub-functionalization mutants have occurred resulting in neither subunit individually having substantial catalytic activity. Here we identify a region still shared by both subunits that affects heat stability and altered kinetic and allosteric properties. (c) Individual amino acid changes affecting AGPase heat stability also affect the kinetic and allosteric properties of the enzyme. This finding is consistent with our previous findings with other amino acid changes. (d) Here we show that a protein motif not involved in 3-PGA binding is present only in AGPase which exhibit significant 3-PGA activation. Importantly, amino acid substitutions in this region affect 3-PGA activation kinetics.

## Author Contributions

SB performed all protein and enzyme investigations. JS performed all DNA mutagenesis work and LH helped in data interpretation. All authors significantly contributed to manuscript preparation.

## Conflict of Interest Statement

The authors declare that the research was conducted in the absence of any commercial or financial relationships that could be construed as a potential conflict of interest.
