# Supplementary material for: Enhancement of Heat Stability and Kinetic Parameters of the Maize Endosperm ADP-Glucose Pyrophosphorylase by Mutagenesis of Amino Acids in the Small Subunit With High B Factors
Source: Front Plant Sci. 2018 Dec 12;9:1849. doi: 10.3389/fpls.2018.01849 (PMC6300691; doi:10.3389/fpls.2018.01849)
Supplement: Supplementary file 1 [file Table_1.DOCX]

**Supp Table 1.** ***K_M_* for ATP and G-1-P (+3-PGA)**

| Mutant | *_KM_* ATP | *_KM_* G-1-P |
| --- | --- | --- |
| wt | 0.075 +/- 0.018 | 0.012 +/- 0.004 |
| E123Q | 0.052 +/- 0.006 | 0.021 +/- 0.004 |
| R331K | 0.088 +/- 0.013 | 0.042 +/- 0.003 |
| R331P | 0.097 +/- 0.015 | 0.041 +/- 0.004 |
| D242H | 0.11 +/- 0.016 | 0.062 +/- 0.005 |
| K402R | 0.079+/-0.015 | 0.046+/-0.003 |
| K403L | 0.082+/-0.017 | 0.02+/-0.002 |
| K403E | 0.069+/-0.012 | 0.03+/-0.003 |
| K403R | 0.075 +/- 0.013 | 0.014 +/- 0.003 |
| L404K | 0.043+/-0.009 | 0.024+/-0.004 |
| L404E | 0.135+/-0.047 | 0.068+/-0.005 |
| L404H | 0.132+/-0.022 | 0.064+/-0.002 |
| E123G, R331K, K402R, L404H | 0.091 +/- 0.008 | 0.063 +/- 0.062 |
| E123G, R331K, K402R **(BT2-BF)** | 0.060 +/- .007 | 0.052 +/- 0.004 |
| E123G, R331K L404H | 0.067 +/- 0.01 | 0.055 +/- 0.007 |
| E123G,R331K | 0.08+/- 0.005 | 0.057 +/-.006 |
|  |  |  |

All assays were performed in 50mM HEPES pH 7.4, 15mM MgCl­_2_ and 2.5 mM 3-PGA. When held constant, ATP and G-1-P were 2mM. ATP concentrations were varied from 0.05-3.0 mM and G-1-P was varied from 0.025 – 1.5 mM.

.
