# Supplementary figures and images for: Enhancement of Heat Stability and Kinetic Parameters of the Maize Endosperm ADP-Glucose Pyrophosphorylase by Mutagenesis of Amino Acids in the Small Subunit With High B Factors
Source: Front Plant Sci. 2018 Dec 12;9:1849. doi: 10.3389/fpls.2018.01849 (PMC6300691; doi:10.3389/fpls.2018.01849)

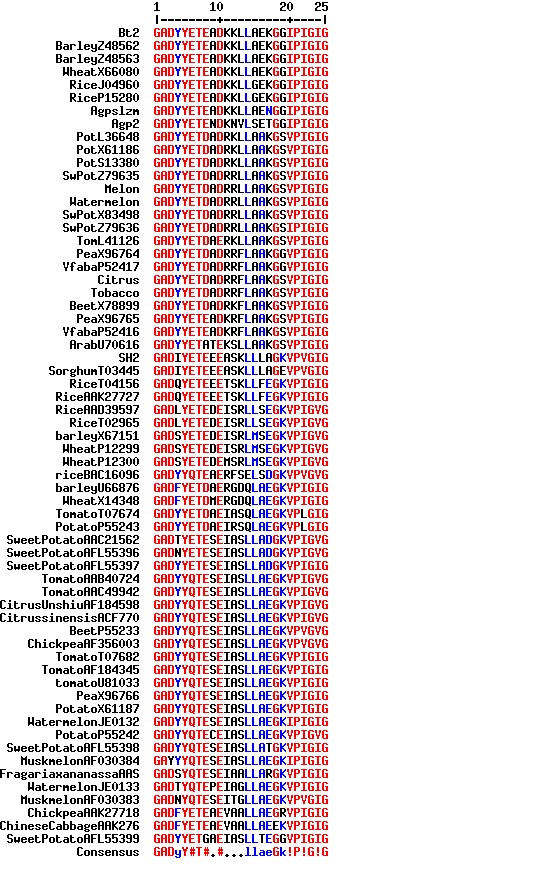

Supplement: Supplementary file 2 [file Image_1.JPEG]
